# Wall shear stress measured by phase contrast cardiovascular magnetic resonance in children and adolescents with pulmonary arterial hypertension

**DOI:** 10.1186/1532-429X-15-81

**Published:** 2013-09-13

**Authors:** Uyen Truong, Brian Fonseca, Jamie Dunning, Shawna Burgett, Craig Lanning, D Dunbar Ivy, Robin Shandas, Kendall Hunter, Alex J Barker

**Affiliations:** 1 Division of Pediatric Cardiology, Children’s Hospital Colorado, Aurora, CO 80045, USA; 2 Department for Bioengineering, University of Colorado, 13123 E. 16th Avenue B100, Aurora, CO 80045, USA; 3 Department of Radiology, Feinberg School of Medicine, Northwestern University, Chicago, IL 60611, USA

**Keywords:** Vessel size, Pulmonary hypertension, Wall shear stress, Cardiovascular magnetic resonance

## Abstract

**Background:**

Pulmonary arterial hypertension (PAH) is a devastating disease with significant morbidity and mortality. At the macroscopic level, disease progression is observed as a complex interplay between mean pulmonary artery pressure, pulmonary vascular resistance, pulmonary vascular stiffness, arterial size, and flow. Wall shear stress (WSS) is known to mediate or be dependent on a number of these factors. Given that WSS is known to promote architectural vessel remodeling, it is imperative that the changes of this factor be quantified in the presence of PAH.

**Methods:**

In this study, we analyzed phase contrast imaging of the right pulmonary artery derived from cardiovascular magnetic resonance to quantify the local, temporal and circumferentially averaged WSS of a PAH population and a pediatric control population. In addition, information about flow and relative area change were derived.

**Results:**

Although the normotensive and PAH shear waveform exhibited a WSS profile which is uniform in magnitude and direction along the vessel circumference at systole, time-averaged WSS (2.2 ± 1.6 vs. 6.6 ± 3.4 dynes/cm^2^, P = 0.018) and systolic WSS (8.2 ± 5.0 v. 20.0 ± 9.1 dynes/cm^2^, P = 0.018) was significantly depressed in the PAH population as compared to the controls. BSA-indexed PA diameter was significantly larger in the PAH population (1.5 ± 0.4 vs. 0.7 ± 0.1 cm/m^2^, P = 0.003).

**Conclusions:**

In the presence of preserved flow rates through a large PAH pulmonary artery, WSS is significantly decreased. This may have implications for proximal pulmonary artery remodeling and cellular function in the progression of PAH.

## Background

Pulmonary arterial hypertension (PAH) is a devastating disease with significant morbidity and mortality [1]. Classified as a subgroup of pulmonary hypertension by the World Health Organization, it includes persistent hypertension of the newborn, hypertension secondary to congenital heart disease, and idiopathic PAH [2]. For all categories, survival rates are poor. Children with idiopathic PAH (comprising 50-60% of those with PAH) have demonstrated 5-year survival rate of 75% in both European studies as well as the US REVEAL (Registry to Evaluate Early and Long Term PAH Disease Management) study [3-5].

Research efforts are complicated by the multifactorial nature of the disease [6-14], involving maladaptive pulmonary wall remodeling and changes in the pulmonary arterial hemodynamic environment [2]. It is postulated that initial injury to endothelial cells in the distal vasculature cause pulmonary artery medial hypertrophy, adventitial thickening, and neo-intimal lesions [15]. A resulting consequence is a progressive increase in pulmonary vascular resistance (PVR) and mean pulmonary artery pressure [15]. Moreover, an elevated mean pulmonary artery pressure is thought to distend the proximal arteries and increase pulmonary vascular stiffness [16,17]. These changes in artery compliance and size can ultimately affect the flow waveform and viscous hemodynamic forces at the artery walls.

A key link in these events is the quantification of how wall shear stress (WSS) - the primary mechanical force affecting cell mechanotransduction - changes in disease conditions [18]. The WSS mechanism is attributed to the observation that high flow pulsatility promotes inflammatory and proliferative cell expression [19,20]. While previous studies have quantified changes in mean pulmonary artery pressure, PVR, pulmonary vascular stiffness, arterial size, flow waveforms, and flow fields in the presence of PAH, WSS has not been evaluated quantitatively in the pediatric PAH patient [6-14]. In addition, there is increasing evidence in the systemic vasculature that low WSS is a promoter of increased wall stiffness and atherogenic vascular states, and is an independent predictor of cardiovascular mortality [21,22]. Since WSS is reported to regulate transcriptional events in vascular remodeling, its quantification may further elucidate the complex etiology involved in PAH. In this study, we aim to quantify the local, temporal and circumferentially averaged WSS by cardiovascular magnetic resonance (CMR) in the right pulmonary artery (RPA) of a pediatric PAH population and a control population. In addition, we assess whether there is a difference in the measured WSS values between these groups.

## Methods

With the approval of the Institutional Review Board at the Children’s Hospital Colorado, all available CMR studies on patients with PAH at our institution were retrospectively analyzed. Patients with prior history of pulmonary artery surgery, right ventricular surgery, pulmonary arterial stenosis, pulmonary valve insufficiency, and chronic thrombolic pulmonary hypertension were excluded. In cases in which multiple studies were performed on a single patient, we chose to analyze the study with the best delineation of the RPA lumen and a high signal-to-noise ratio phase contrast data. Patients with a poor slice position through the RPA, sternal wire susceptibility artifacts, or poorly defined artery lumen boundaries were excluded (*n* = 5). For the normotensive group, we included patients who were referred for syncope with normal CMR, vascular rings, and mild left-sided cardiac anomalies that include non-stenotic bicuspid aortic valve and uncomplicated coarctation repair. Demographic data for control versus PAH subjects are shown in Table 1.

**Table 1:** Demographics and flow quantification of PAH study patients

	***Control (n = 4)***	***PAH (n = 25)***	***P***
***Age, (y)***	19 ± 12	12 ± 5	0.3
***Sex, (M:F)***	2:2	11:14	
***BSA (m***^***2***^***)***	1.7 ± 0.7	1.2 ± 0.3	0.14
***RPA diam (cm)***	1.2 ± 0.5	1.8 ± 0.7	0.19
***RPA/ BSA (cm/m***^***2***^***)***	0.7 ± 0.1	1.5 ± 0.4	0.003
***RAC (%)***	32 ± 18	21 ± 11	0.15
***Vmean***_***t_ avg ***_***(cm/s)***	30 ± 12	17 ± 11	0.05
***Vmean***_***systole ***_***(cm/s)***	83 ± 23	*58 ± 34*	0.07
***Qavg (L/min)***	2.6 ± 1.2	2.4 ± 1.0	0.55
***Qsys (L/min)***	9.0 ± 4.4	8.9 ± 3.7	0.68
***V***_***max***_***(m/s)***	1.3 ± 0.7	*0.8 ± 0.5*	0.14
***RF(%)***	0.9 ± 0.9	1.1 ± 2.0	0.47
***WSS***_***t_avg***_***(dyne/cm2)***	−6.6 ± 3.4	−2.2 ± 1.6	0.018

Values are mean ± SD; PAH = pulmonary arterial hypertension; BSA = body surface area (m^2^); RPA = right pulmonary artery diameter (cm); RPA/BSA = normalized RPA diameter (cm/m^2^); RAC = Relative Area Change (%); Vmean_t_avg_ = time-averaged mean velocity over vessel cross-section; Vmean_systole_ = systolic mean velocity over vessel cross-section; Q_avg_ = average flow rate (L/min); Q_sys_ = peak systolic flow rate (L/min); RF = regurgitant fraction (%); V_max_ = max velocity (m/s); WSS_t_avg_ = circumferentially averaged WSS over cardiac cycle.

### CMR Protocol

A fast low-angle shot gradient echo sequence was used to obtain retrospectively gated tissue intensity and phase velocity maps encoded in the through-plane direction (1.5 T Siemens Magnetom Avanto). Double oblique imaging slices were positioned between the proximal and the first branch of the RPA, orthogonal to the vessel long axis. A typical sequence used temporal resolution of 14–28 ms, echo times of 2.2-3.5 ms, and a flip angle of 25˚. Depending on patient size and field of view, the cross sectional pixel resolution was 0.82 × 0.82 to 1.56 × 1.56 mm with slice thicknesses of 5 mm. Velocity encoding values were adjusted according to the maximum velocities encountered during scout sequences to optimize the velocity map resolution (typical values ranged from 200 to 250 cm/s).

### Post-processing and RPA size

**Figure 1:**
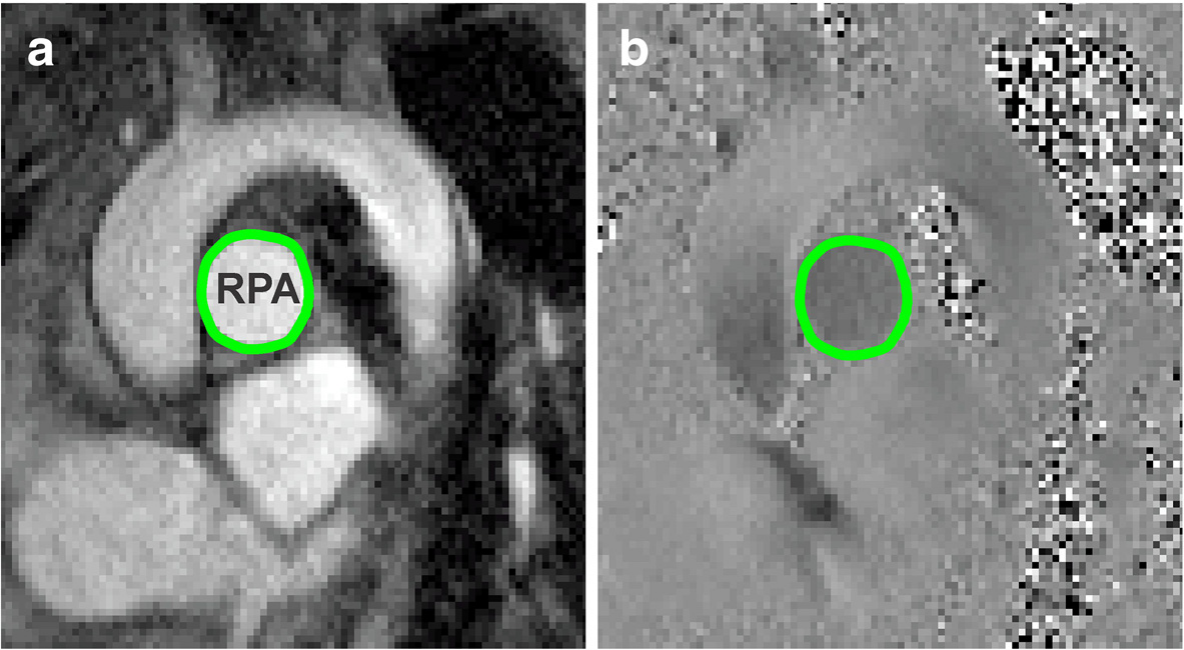
**Phase-contrast imaging through the right pulmonary artery.** Segmented **(a)** intensity and **(b)** phase contrast images (RPA lumen shown in green).

**Figure 2:**
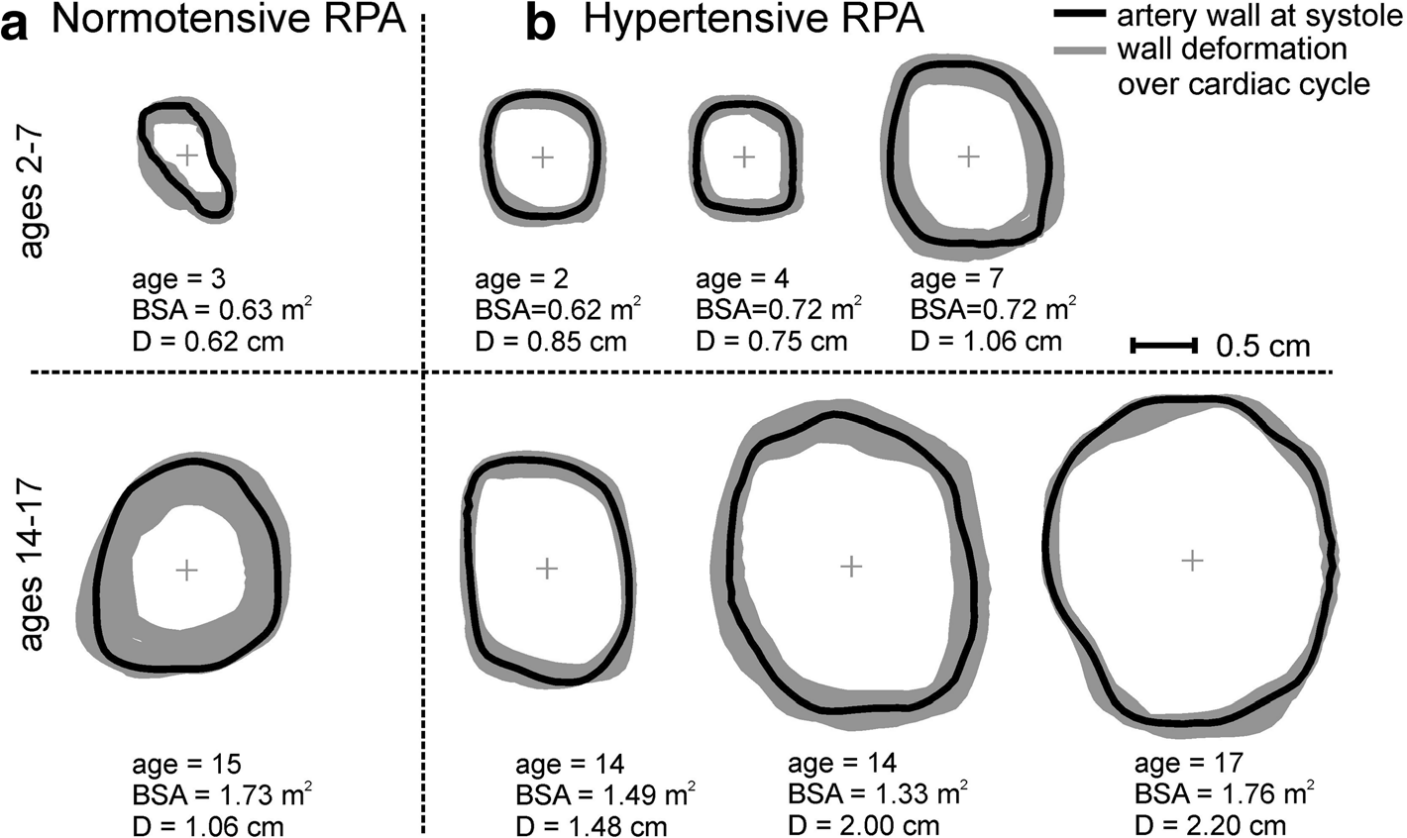
**RPA area change and relative size.** Normotensive **(a)** and PAH subjects **(b)** are shown with the calculated diameter (D), as back-calculated from diastolic cross-sectional area.

The RPAs were carefully segmented (Figure 1) over the cardiac cycle using a semiautomatic level-set method (Segment, Medviso), and exported to a previously described Matlab (Mathworks, Inc., Natick, MA) program developed to record time-resolved RPA diameter measurements, blood flow (positive, negative and net), peak blood velocity (*V*_max_), and the temporal and spatial WSS fields [23,24]. Pulmonary artery diameters were calculated by evaluating the ROI area at diastole and back calculating the effective diameter, assuming a circular cross section (Figure 2). These were normalized by the body surface area, which was calculated by Haycock’s method to obtain an indexed artery size for interpatient comparison [25]. The relative area change (RAC) of the arterial wall was calculated using the relation RAC = 100*[(A_max_-A_min_)/A_min_], where A_max_ and A_min_ represent the maximum and minimum measured cross-sectional areas.

### Flow & WSS quantification parameters

The average flow rate (*Q*_avg_), flow rate at systole (*Q*_sys_), and regurgitant fraction (*RF*) were calculated according to convention. To facilitate the calculation of the shear fields and remove RPA bulk motion, the images were cropped to the RPA size and transformed from Cartesian to polar coordinates using oversampling (sampling at the vessel wall was set to double the original resolution) and bicubic interpolation to retain image integrity at the region of interest (ROI) edges. As previously described, a linearized axial shear stress approximation was used to obtain the axial RPA shear field over all time-steps [23]. The axial WSS was then sampled at 45° increments along the RPA lumen to create a localized, position specific, waveform over the cardiac cycle. These positions are referred to in the text and figures as S, A, I, P representing the superior, anterior, inferior, and posterior RPA lumen locations. WSS values averaged over the circumference of the artery wall at flow systole (*WSS*_*systole*_) and averaged over the cardiac cycle (*WSS*_*t_avg*_) were also calculated as described previously [23].

### Measurement reliability

In a subset of 10 random PAH and normotensive patients, *WSS*_*t_avg*_ and *WSS*_*systole*_ were calculated from 2 separate segmentations of the RPA, to determine intra-observer variability (JD). Interobserver error was calculated from RPA segmentations performed by two blinded researchers (AB and JD), and assessed with the values for *WSS*_*t_avg*_ and *WSS*_*systole*_.

### Statistical analysis

All parameters are expressed as mean ± standard deviation. A Wilcoxon rank sum test was used to determine if parameter means were significantly different, where p < 0.05 was considered statistically significant. A regression analysis similar to that previously reported [26] for healthy children was used to model the normal RPA diameter as a function of BSA (i.e. D = a*BSA^*0.5*^) and 95% confidence intervals were calculated to visualize deviation from normal values.

## Results

Twenty-five PAH patients and 4 normotensive subjects with valid CMR images were analyzed. The mean age for the PAH group was 12 ± 5 years (ranging from 2 to 20 years), with a median of 11.5 years. The mean age for the normotensive patient group was 19 ± 12 (ranging from 3 to 31 years). A summary of the demographics and bulk flow quantification parameters are shown Table 1.

**Figure 3:**
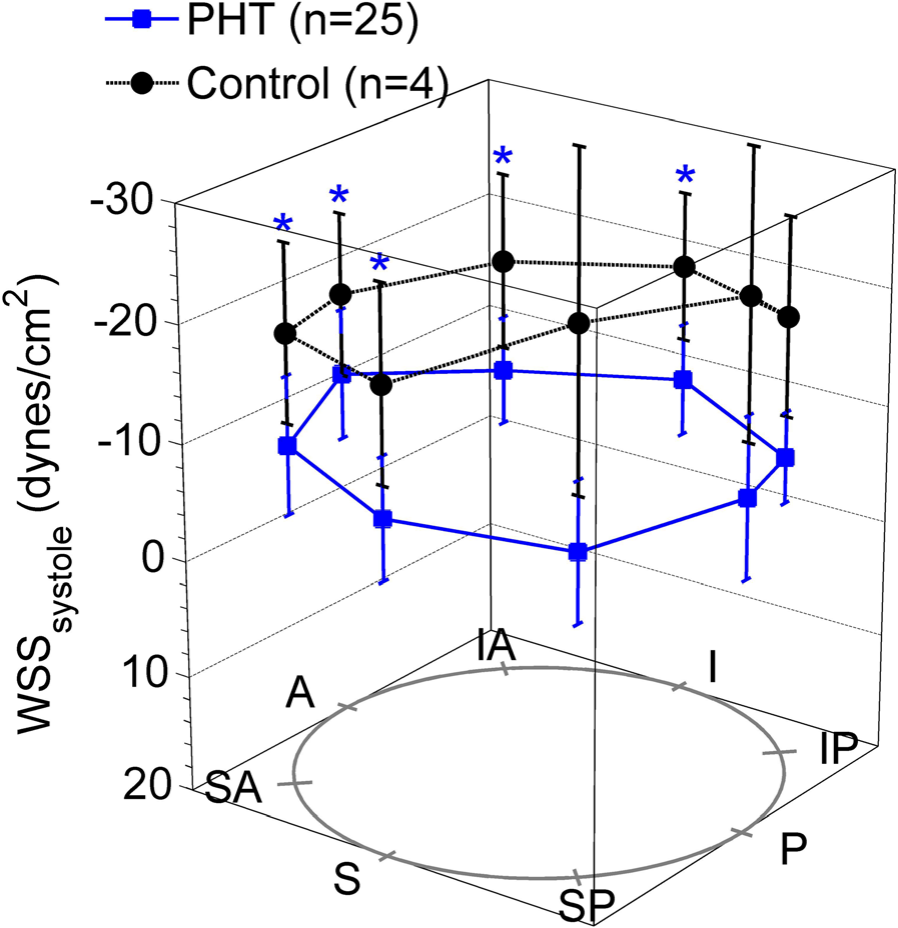
**Regional wall shear stress in systole in the normotensive population (n = 4) compared to the PAH population (n = 25).** P < 0.05 is indicated by ‘*’.

**Figure 4:**
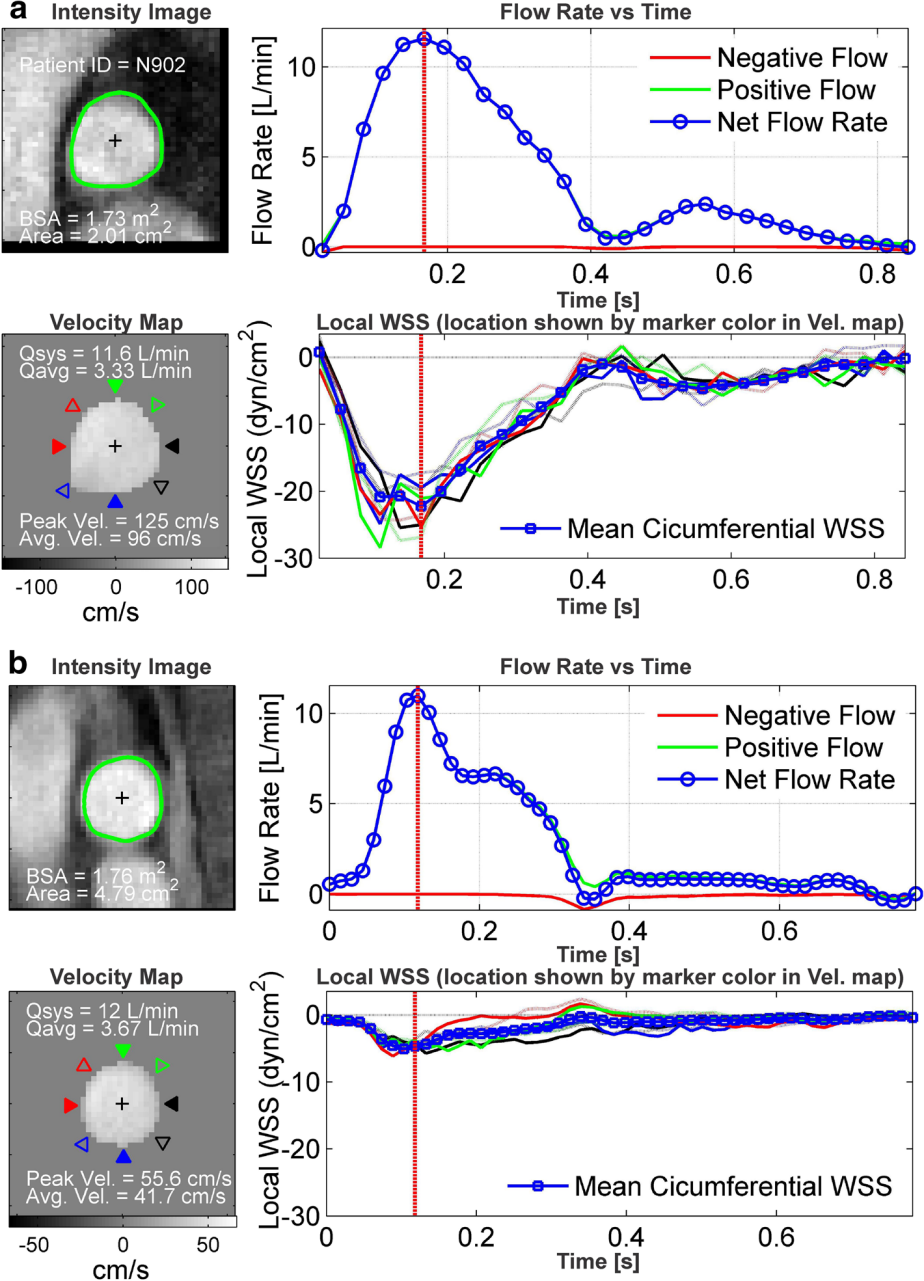
**PAH patient morphology and WSS summary.** Morphology, velocity, flow, and WSS summary for **(a)** an example normotensive subject and **(b)** an example PAH patient. The subjects are and BSA matched. Note the drastic overall WSS reduction in the PAH patient.

**Figure 5:**
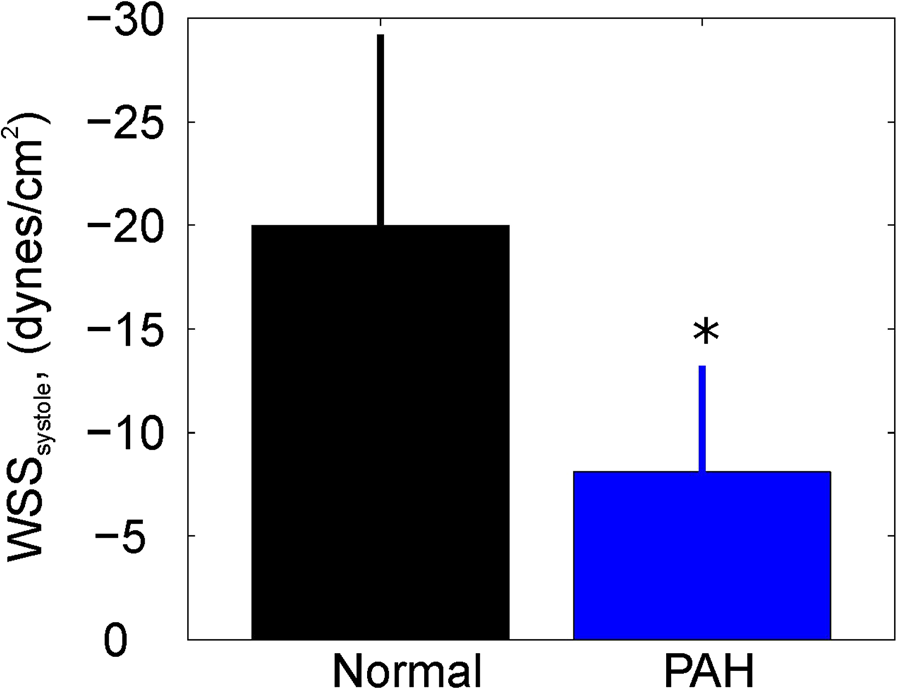
**Cross-sectional average of *WSS***_***systole***_ in PAH patients as compared to normotensive controls (‘*’ indicates P < 0.05). P < 0.05 is indicated by ‘*’.

The normalized RPA size was significantly larger in the PAH population (p = 0.003) and evidence of a reduction in RAC was observed (Figure 2), although not statistically significant (p = 0.15). Regional *WSS*_*systole*_ and the temporal WSS waveforms (Figures 3 and 4), were found to be markedly different. Figure 4 shows an example of the flow and regional WSS waveforms for a PAH patient and a normotensive subject. Both the normotensive and PAH shear waveform exhibit a WSS profile which is uniform in magnitude and direction along the vessel circumference at systole. However, the magnitude of the regional *WSS*_*systole*_ differed drastically, as illustrated in the population means between the two cohorts for *WSS*_*t_avg*_*and WSS*_*systole*_ (Table 1 and Figure 5).

Intraobserver variability analysis revealed that the percentage error was 3.9% for *WSS*_*t_avg*_ and 5.1% for *WSS*_*sys*_. Interobserver variability was 2.7% for *WSS*_*t_avg*_ and 2.8% for *WSS*_*sys*_.

## Discussion

There has been a paradigm shift away from the idea that pulmonary hypertension is merely a disease of the distal vessels [17,27]. Recent evidence has emerged warranting further examination of proximal pulmonary artery remodeling in the disease pathway [17,27-29]. Proximal arterial dilation is a result of the second phase of proximal vascular stiffening in PAH, in which elastin function is lost, and the resulting dilated vessels must carry pressure load and maintain nearly constant diameter over the cardiac cycle (the geometric effects are observed via CMR in Figure 2) [17,30]. This increases RV load substantially and removes the dual-stage action of the right ventricle-pulmonary artery pump, requiring the RV to work even harder [31]. Such changes profoundly alter proximal flow dynamics, but it is uncertain how they affect overall pulmonary mechanotransduction as well as distal flow [17]. Alteration in pulsatile flow promotes inflammatory and proliferative cell expression [19,20]. Changes in WSS in the proximal arteries caused by changes in flow may be key to understanding the disease development. In this study, we are the first to describe depressed WSS values in a pediatric PAH population.

Currently, the reference standard for diagnosing, monitoring, and evaluating therapeutic response in the presence of PAH is cardiac catheterization. The invasive nature of this procedure has led clinicians to look towards non-invasive imaging modalities for similar information. While echocardiography has multiple advantages, CMR allows accurate assessment of ventricular volume, mass, function, and evidence of myocardial fibrosis. CMR also has the potential to provide detailed WSS measurements, such as the case in atherosclerotic and aneurysm models [18,32,33], although this has not been evaluated thoroughly in the pediatric patient. WSS, which is determined by the velocity gradient, cannot be measured by ultrasound Doppler modalities, and approximations using a Poiseuille (steady) profile have been shown to contain significant error. Previous efforts quantifying WSS in children have used computational fluid dynamics to model the effect of PAH on WSS [34] and phase contrast (PC)-CMR has been used to quantify WSS in healthy and pre- and post-operative Fontan patients [35]. Tang et al. [36]. described WSS using computational fluid dynamics in PAH patients; however all but one was older than 18 years of age.

**Figure 6:**
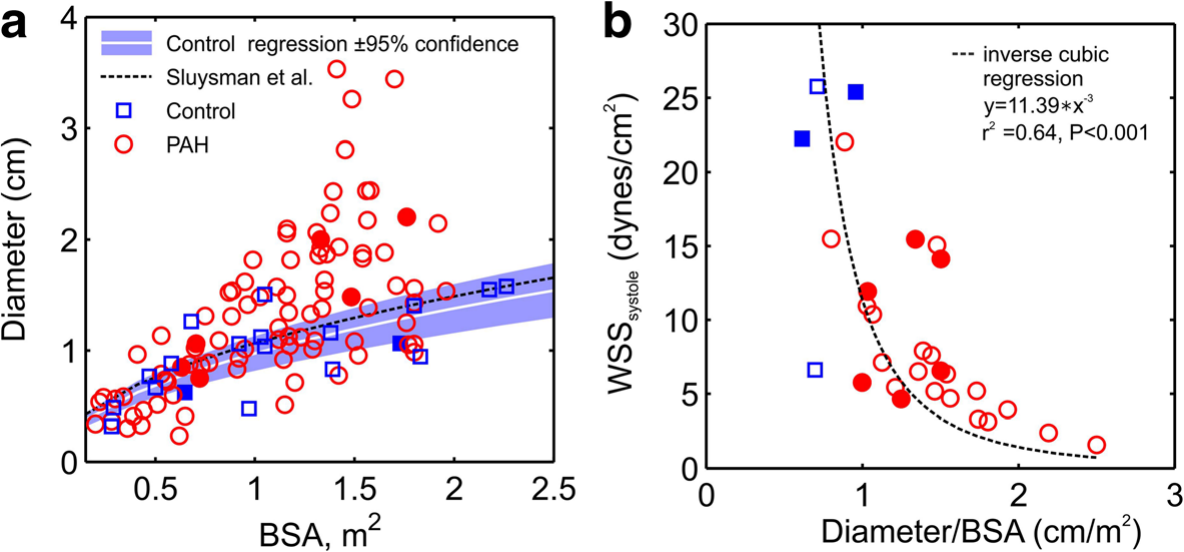
**Relationship between right pulmonary arterial diameter and WSS with BSA in controls and subjects with pulmonary arterial hypertension. (a)** RPA diameter and WSS measurements in systole demonstrate a BSA dependence in both cohorts. The white line indicates the regression model (y = ax^0.5^) for the control group, with the blue shaded region indicating the 95% confidence region; the dashed line indicates results (n = 496) from Sluysman et al. [26]. **(b)** WSS decreases rapidly as a function of the BSA-indexed RPA diameter. Solid markers indicate subjects with cross-sectional profiles plotted in Figure 2. The regression line in **(b)** is an inverse cubic regression, reflecting the inverse proportional relationship between WSS and the vessel radius cubed. Note: diameter measurements are augmented with data collected in previous echo-based studies [33]; WSS measurements are from CMR-only.

We postulate that the observed proximal WSS decrease is directly related to the increased size of the proximal pulmonary arteries in the presence of PAH. In preliminary studies, we used Color M-mode tissue Doppler echocardiography [27,31] to evaluate the differences in RPA diameter in PAH children versus in normotensive children. Figure 6a depicts a combination of the previously collected RPA diameter data (n = 66 PAH and n = 15 Control) and CMR data collected in this study (as plotted against BSA). The drastic departure of the PAH children from the 95% confidence bounds is evident with increasing BSA. An overlay of normal RPA diameters from Sluysman et al. [26] (n = 496) reinforces this observation (Figure 6a, dashed line); additionally, Schiebler et al. [37] recently reported this enlargement trend in adult PAH. Given that compared to the normal subjects, PAH children had similar peak and average flow rates (Table 1) and that their RPA diameter was larger, the result will be decreased WSS, as was observed for both *WSS*_*t_avg*_ and *WSS*_*systole*_. Indeed, a strong trend toward decreasing WSS is seen as a function of the indexed RPA radius cubed (Figure 6b), which corresponds to the idealized pipe flow relationship between flow and diameter (i.e. *WSS* α [viscosity*flow/Diameter^3^] [23]. These results suggest that dilatation is a progressive process in pediatric PAH and the consequence of maintaining equal flow through a larger artery is a decrease in WSS.

The results from our study are in concordance with previous efforts. Pelc et al. initially noted that main pulmonary artery flow at peak systole in PAH patients was more spatially heterogeneous and demonstrated a greater percentage of retrograde flow [38]. Building on this observation, a number of researchers have quantified bulk flow hemodynamics in the pulmonary arteries. Among them, Ley performed a detailed study of 25 PAH patients and 25 volunteers to compare the peak velocity, average flow, time-to-peak velocity, velocity rise gradient, and pulmonary distensibility [11]. In comparison to the volunteers, the PAH patients showed significantly reduced pulmonary velocities (P = 0.002), blood flow (P = 0.002), and pulmonary distensibility (P = 0.008). The patients also showed a shorter time-to-peak velocity (P < 0.001) with a steeper velocity rise gradient (P = 0.002), which is consistent with higher pulsatility and increased higher harmonics of impedance.

In addition, Morgan et al. has looked at the WSS forces in healthy pulmonary arteries [39]. As mentioned previously, WSS was found to be relatively constant throughout the cardiac cycle and axial, circumferential, and radial WSS averaged approximately 7 dynes/cm^2^. There was no statistical difference between the vessels for circumferential or radial WSS. Our study extends these previous investigations to determine the axial WSS forces in pediatric PAH patients. Table 1 summarizes the population demographics. Normalized RPA size, *WSS*_*t_avg*_ and *WSS*_*systole*_ were all found to differ significantly. The difference in the calculated WSS values between these two patient groups appears to be associated with artery dilation, as seen in the artery morphology comparison of matched patients in Figure 2[11]. A case highlighting the effect artery size may have on WSS is illustrated by removing systolic flow as a variable between the BSA-matched normotensive and PAH patients shown in Figure 4. These patients have a similar systolic flow rate of 11.6 and 12.0 L/min - with an indexed RPA size of 1.1 cm^2^/m^2^ and 2.7 cm^2^/m^2^, respectively. We postulate that as a result of this arterial size difference in the normotensive patient, and thus a change in spatial velocity gradients, the peak WSS magnitude is more than 4.5 times than that of the PAH patient.

Gan used area distensibility and relative area change to predict mortality in PAH [7]. Besides finding that the cross-sectional area was much greater and the relative area change much less in PAH patients, relative area change was found to be the strongest predictor of mortality over distensibility, independent of pulse pressure. As well as measuring flow, pulse wave velocity, WSS, and distensibility in the proximal pulmonary arteries, a number of authors have observed highly inhomogeneous cross-sectional area flow profiles and retrograde flow in the main pulmonary artery of PAH patients [6,10,12]. Even though no correlation between retrograde flow and the degree of PAH has been found [10,12], these observations suggest that PAH may influence some form of flow derangement in the right ventricular outflow tract [14]. Neither echocardiography nor 2D PC-CMR has shown any conclusive findings. However, Reiter et al. used time-resolved 3D velocity information to visualize this flow anomaly and found a vortex consistently formed in the main pulmonary artery of PAH patients, just below the RPA. It was found that the time persistence of this vortex was correlated to the degree of hypertension as measured by mean pulmonary artery pressure [14].

A number of concomitant factors contribute to changes in the pulmonary arterial pressure of PAH patients, including increased PVR, increased blood flow due to shunting, and alterations in pulmonary vascular stiffness. The influence of pathologic pulmonary pressures, PVR, and pulmonary vascular stiffness on proximal morphologic and hemodynamic factors may include pulmonary arterial dilatation, flow waveform alterations, and WSS alterations [40]. Since WSS is reported to regulate transcriptional events in vascular remodeling, its quantification is important in order to model the complex etiology involved in PAH. PC-CMR is a promising tool for this application. It has been demonstrated to accurately and non-invasively quantify large artery spatial velocity fields, allowing for the measurement of spatial and temporal WSS [23]. Shear in PAH resembles an "atherosclerotic/aneurysm phenotype" in that mean shear is greatly reduced proximally, potentially altering endothelial function and mechanotransduction in the large vessels. Our group and others have shown that altered flow profiles (and thus, altered shear profiles) result in cellular expression of inflammatory cytokines from more distal vessels [19,20]. This work is part of an ongoing effort to establish a chain of events that eventually lead to worsening PAH; that is, dilation of vessels and distensibility lead to altered shear, which leads to altered cell expression, causing medial hypertrophy and endothelium proliferation, elevating PVR, and ultimately culminating in right ventricular failure.

### Limitations

Limitations to the study include those inherent to a retrospective study. We recognize that our CMR control population is small as this number is severely restricted by availability of RPA PC-CMR sequences in children without right heart disease. To address this, we included echocardiography results from healthy controls with no known heart disease, which shows significant differences in pulmonary arterial vasculature compared to pediatric PAH population. For the PC-CMR sequences, we included children with mild left-sided heart disease with no evidence of abnormal pulmonary artery pressure. However, we recognize the potential for mild pulmonary arterial flow abnormality caused by upstream anatomic anomalies. Efforts to enroll healthy children with no cardiac structural disease are ongoing.

## Conclusion

We have demonstrated the use of PC-CMR to quantify hemodynamic WSS in PAH patients. Pulmonary artery dilation occurs at a significantly faster rate in PAH patients, while systolic flow does not vary between populations. Thus, the consequence of maintaining similar net flow through a larger artery is a WSS decrease in PAH patients. This may have implications for proximal pulmonary artery remodeling and cellular function.

## Abbreviations

CMR: Cardiovascular magnetic resonance; PAH: Pulmonary arterial hypertension; PC: Phase-contrast; PVR: Pulmonary vascular resistance; RPA: Right pulmonary artery; RV: Right ventricle; WSS: Wall shear stress.

## Competing interests

The authors declare that they have no competing interests.

## Authors’ contributions

UT- writing manuscript, study design, subject scanning, data selection. BF- Study design, subject recruitment, subject scanning. JD- data selection, statistical analysis. SB- statistical analysis. CL-design of analysis program. DI-study design. RS-study design. KH- Study design, statistical analysis, editing and writing manuscript. AB- study design, data analysis, editing and writing manuscript. All authors read and approved the final manuscript.

## Authors’ information

Uyen Truong and Brian Fonseca are co-first authors.

Kendall Hunter and Alex J Barker are co-senior authors.
